# Supplementary material for: Strategic prioritization of blockchain diffusion factors by an integrated fuzzy Delphi-Dombi LMAW model: A case study in Bangladesh healthcare sectors
Source: PLoS One. 2026 May 14;21(5):e0348578. doi: 10.1371/journal.pone.0348578 (PMC13175360; doi:10.1371/journal.pone.0348578)
Supplement: S1 File — This file contains S1 Table (Identified factors), S2 Table (Collected Data from the experts on identifying major factors), S3 Table (Defuzzification of the expert’s opinion), and S4 Table (Data of the Factors Ratio Vectors for Healthcare A from the remaining five experts). (DOCX) [file pone.0348578.s001.docx]

### Survey Questionnaires for fuzzy Delphi method**:**

There are total 26 blockchain diffusion factors listed in the following table that affect the Supply Chain in Healthcare Industries. The comprehensive list of the initial 26 Blockchain Diffusion Factors identified for expert evaluation in the Fuzzy Delphi Method is shown in S1 Table. In your opinions, rate the factors below:

**S1 Table. Identified factors**

| **Sustainability Aspects** | **Blockchain Diffusion Factors** | **Ratings**  **(0 to 9)** |
| --- | --- | --- |
| Technological factors | 1. Blockchain scalability |  |
|  | 2. Cybersecurity risks |  |
|  | 3. Interoperability with legacy systems |  |
|  | 4. Integration with IT infrastructure |  |
|  | 5. Standardization issues |  |
|  | 6. Smart contract automation |  |
|  | 7. Traceability |  |
| Organizational factors | 8. Top management support |  |
|  | 9. Organizational readiness |  |
|  | 10. Implementation cost |  |
|  | 11. Resistance to change |  |
|  | 12. Lack of ROI clarity |  |
|  | 13. Lack of practical use cases |  |
| Managerial factors | 14. Trust among stakeholders |  |
|  | 15. Transparency and immutability |  |
|  | 16. Disintermediation (no middlemen) |  |
|  | 17. Perceived benefits (efficiency, speed) |  |
| Environmental factors | 18. Regulatory and legal clarity |  |
|  | 19. Competitive pressure |  |
|  | 20. Vendor readiness |  |
|  | 21. Innovation pressure |  |
|  | 22. Performance expectancy |  |
| Individual factors | 23. Lack of awareness |  |
|  | 24. Trust in technology |  |
|  | 25. Effort expectancy |  |
|  | 26. Social influence |  |
| ****Please provide any suggestion on healthcare supply chain if there are any challenges on your opinion?** | |  |

The raw expert input data on the 26 Blockchain Diffusion Factors using Triangular Fuzzy Numbers is shown in S2 Table.

**S2 Table. Collected Data from the experts on identifying major factors**

| **Factor** | **Expert 1** | **Expert 2** | **Expert 3** | **Expert 4** |
| --- | --- | --- | --- | --- |
| Blockchain scalability | (10, 10, 10) | (7, 8, 9) | (6, 7, 8) | (10, 10, 10) |
| Cybersecurity risks | (2, 3, 4) | (2, 3, 4) | (1, 2, 3) | (3, 4, 5) |
| Interoperability with legacy systems | (7, 8, 9) | (8, 9, 10) | (7, 8, 9) | (8, 9, 10) |
| Integration with IT infrastructure | (10, 10, 10) | (7, 8, 9) | (6, 7, 8) | (6, 7, 8) |
| Standardization issues | (6, 7, 8) | (6, 7, 8) | (5, 6, 7) | (8, 9, 10) |
| Smart contract automation | (5, 6, 7) | (7, 8, 9) | (7, 8, 9) | (6, 7, 8) |
| Traceability | (8, 9, 10) | (8, 9, 10) | (7, 8, 9) | (5, 6, 7) |
| Top management support | (10, 10, 10) | (7, 8, 9) | (10, 10, 10) | (10, 10, 10) |
| Organizational readiness | (7, 8, 9) | (10, 10, 10) | (8, 9, 10) | (10, 10, 10) |
| Implementation cost | (6, 7, 8) | (6, 7, 8) | (8, 9, 10) | (10, 10, 10) |
| Resistance to change | (5, 6, 7) | (8, 9, 10) | (7, 8, 9) | (10, 10, 10) |
| Lack of ROI clarity | (5, 6, 7) | (7, 8, 9) | (7, 8, 9) | (6, 7, 8) |
| Lack of practical use cases | (0, 0, 1) | (3, 4, 5) | (0, 0, 1) | (1, 2, 3) |
| Trust among stakeholders | (10, 10, 10) | (10, 10, 10) | (8, 9, 10) | (7, 8, 9) |
| Transparency and immutability | (7, 8, 9) | (8, 9, 10) | (6, 7, 8) | (6, 7, 8) |
| Disintermediation | (8, 9, 10) | (6, 7, 8) | (7, 8, 9) | (5, 6, 7) |
| Perceived benefits | (5, 6, 7) | (10, 10, 10) | (5, 6, 7) | (6, 7, 8) |
| Regulatory and legal clarity | (8, 9, 10) | (7, 8, 9) | (6, 7, 8) | (10, 10, 10) |
| Competitive pressure | (8, 9, 10) | (8, 9, 10) | (7, 8, 9) | (5, 6, 7) |
| Vendor readiness | (5, 6, 7) | (5, 6, 7) | (8, 9, 10) | (8, 9, 10) |
| Innovation pressure | (6, 7, 8) | (7, 8, 9) | (5, 6, 7) | (5, 6, 7) |
| Performance expectancy | (2, 3, 4) | (0, 0, 1) | (3, 4, 5) | (3, 4, 5) |
| Lack of awareness | (6, 7, 8) | (5, 6, 7) | (6, 7, 8) | (8, 9, 10) |
| Trust in technology | (5, 6, 7) | (8, 9, 10) | (10, 10, 10) | (8, 9, 10) |
| Effort expectancy | (0, 0, 1) | (0, 0, 1) | (2, 3, 4) | (1, 2, 3) |
| Social influence | (10, 10, 10) | (6, 7, 8) | (7, 8, 9) | (7, 8, 9) |

S3 Table shows the Aggregated Fuzzy Scores, Defuzzified Scores, and the final decision on the 26 factors using the Fuzzy Delphi consensus threshold.

**S3 Table. Defuzzification of the expert’s opinion**

| **Factor** | **Aggregated TFN** | **Defuzzified Score** | **Decision** |
| --- | --- | --- | --- |
| Blockchain scalability | (6.00, 8.65, 10.00) | 8.217 | Accepted |
| Cybersecurity risks | (1.00, 2.91, 5.00) | 2.971 | Rejected |
| Interoperability with legacy systems | (7.00, 8.49, 10.00) | 8.495 | Accepted |
| Integration with IT infrastructure | (6.00, 7.91, 10.00) | 7.971 | Accepted |
| Standardization issues | (5.00, 7.17, 10.00) | 7.391 | Accepted |
| Smart contract automation | (5.00, 7.20, 9.00) | 7.067 | Accepted |
| Traceability | (5.00, 7.90, 10.00) | 7.632 | Accepted |
| Top management support | (7.00, 9.46, 10.00) | 8.819 | Accepted |
| Organizational readiness | (7.00, 9.21, 10.00) | 8.737 | Accepted |
| Implementation cost | (6.00, 8.15, 10.00) | 8.05 | Accepted |
| Resistance to change | (5.00, 8.11, 10.00) | 7.702 | Accepted |
| Lack of ROI clarity | (5.00, 7.20, 9.00) | 7.067 | Accepted |
| Lack of practical use cases | (0.00, 0.00, 5.00) | 1.667 | Rejected |
| Trust among stakeholders | (7.00, 9.21, 10.00) | 8.737 | Accepted |
| Transparency and immutability | (6.00, 7.71, 10.00) | 7.902 | Accepted |
| Disintermediation | (5.00, 7.42, 10.00) | 7.472 | Accepted |
| Perceived benefits | (5.00, 7.09, 10.00) | 7.362 | Accepted |
| Regulatory and legal clarity | (6.00, 8.43, 10.00) | 8.142 | Accepted |
| Competitive pressure | (5.00, 7.90, 10.00) | 7.632 | Accepted |
| Vendor readiness | (5.00, 7.35, 10.00) | 7.449 | Accepted |
| Innovation pressure | (5.00, 6.70, 9.00) | 6.9 | Accepted |
| Performance expectancy | (0.00, 0.00, 5.00) | 1.667 | Rejected |
| Lack of awareness | (5.00, 7.17, 10.00) | 7.391 | Accepted |
| Trust in technology | (5.00, 8.35, 10.00) | 7.783 | Accepted |
| Effort expectancy | (0.00, 0.00, 4.00) | 1.333 | Rejected |
| Social influence | (6.00, 8.18, 10.00) | 8.06 | Accepted |

Threshold value is calculated by using Eq. (4), which is 179.6/26 = 6.9
So, from the result, 22 among of 26 factors are accepted for further analysis.

The data of the Factors Ratio Vectors for Healthcare A from the remaining five experts (E6-E10) is shown in S4 Table.

**S4 Table. Data of the Factors Ratio Vectors for Healthcare A from the remaining five experts**

| **Factors** | **E6** | **E7** | **E8** | **E9** | **E10** |
| --- | --- | --- | --- | --- | --- |
| OF1 | (13.33,18,22.5) | (13.33,18,22.5) | (10,14,20) | (13.33,18,22.5) | (13.33,18,22.5) |
| OF2 | (10,14,20) | (13.33,18,22.5) | (11.67,16,22.5) | (11.67,16,22.5) | (13.33,18,22.5) |
| OF3 | (11.67,16,22.5) | (11.67,16,22.5) | (13.33,18,22.5) | (10,14,20) | (11.67,16,22.5) |
| OF4 | (13.33,18,22.5) | (13.33,18,22.5) | (11.67,16,22.5) | (13.33,18,22.5) | (13.33,18,22.5) |
| OF5 | (10,14,20) | (10,14,20) | (10,14,20) | (10,14,20) | (11.67,16,22.5) |
| TF1 | (13.33,18,22.5) | (13.33,18,22.5) | (11.67,16,22.5) | (11.67,16,22.5) | (10,14,20) |
| TF2 | (11.67,16,22.5) | (11.67,16,22.5) | (11.67,16,22.5) | (13.33,18,22.5) | (11.67,16,22.5) |
| TF3 | (10,14,20) | (13.33,18,22.5) | (8.33,12,17.5) | (10,14,20) | (8.33,12,17.5) |
| TF4 | (11.67,16,22.5) | (13.33,18,22.5) | (8.33,12,17.5) | (13.33,18,22.5) | (11.67,16,22.5) |
| TF5 | (3.33,6,10) | (8.33,12,17.5) | (11.67,16,22.5) | (13.33,18,22.5) | (13.33,18,22.5) |
| TF6 | (6.67,10,15) | (11.67,16,22.5) | (13.33,18,22.5) | (13.33,18,22.5) | (11.67,16,22.5) |
| PF1 | (11.67,16,22.5) | (10,14,20) | (11.67,16,22.5) | (10,14,20) | (11.67,16,22.5) |
| PF2 | (3.33,6,10) | (10,14,20) | (11.67,16,22.5) | (11.67,16,22.5) | (10,14,20) |
| PF3 | (8.33,12,17.5) | (8.33,12,17.5) | (10,14,20) | (13.33,18,22.5) | (13.33,18,22.5) |
| PF4 | (11.67,16,22.5) | (13.33,18,22.5) | (10,14,20) | (11.67,16,22.5) | (11.67,16,22.5) |
| GF1 | (11.67,16,22.5) | (8.33,12,17.5) | (11.67,16,22.5) | (13.33,18,22.5) | (13.33,18,22.5) |
| GF2 | (10,14,20) | (13.33,18,22.5) | (13.33,18,22.5) | (11.67,16,22.5) | (6.67,10,15) |
| GF3 | (11.67,16,22.5) | (13.33,18,22.5) | (13.33,18,22.5) | (13.33,18,22.5) | (10,14,20) |
| GF4 | (11.67,16,22.5) | (8.33,12,17.5) | (10,14,20) | (13.33,18,22.5) | (10,14,20) |
| EF1 | (8.33,12,17.5) | (10,14,20) | (11.67,16,22.5) | (10,14,20) | (6.67,10,15) |
| EF2 | (11.67,16,22.5) | (13.33,18,22.5) | (13.33,18,22.5) | (8.33,12,17.5) | (8.33,12,17.5) |
| EF3 | (8.33,12,17.5) | (11.67,16,22.5) | (11.67,16,22.5) | (11.67,16,22.5) | (11.67,16,22.5) |
