# Supplementary material for: Strategic prioritization of blockchain diffusion factors by an integrated fuzzy Delphi-Dombi LMAW model: A case study in Bangladesh healthcare sectors
Source: PLoS One. 2026 May 14;21(5):e0348578. doi: 10.1371/journal.pone.0348578 (PMC13175360; doi:10.1371/journal.pone.0348578)
Supplement: Attachment — Submitted filename: Response to Reviewers.docx [file pone.0348578.s003.docx]

**MANUSCRIPT INFORMATION**

**Manuscript Number: PONE-D-25-37578**

**Manuscript Title:** Strategic Prioritization of Blockchain Diffusion Factors by an Integrated Fuzzy Delphi-Dombi LMAW Model: A Case Study in Bangladesh Healthcare Sectors.

**Journal:** PLOS ONE

**Authors:** Nasif Morshed^a^, Raad Shahamat Labib^a^, Mushfiqur Rahman^a^, Kazi Md. Tanvir Anzum*^a^

**Corresponding Author:** Kazi Md. Tanvir Anzum

**Email:** [tanvir@iem.kuet.ac.bd](mailto:tanvir@iem.kuet.ac.bd)

______________________________________________________________________________

We appreciate the constructive feedback provided by the editors and reviewers of PLOS ONE, which helped to make the manuscript better. The authors have prepared a revised manuscript that takes into account all of the reviewers' and editor comments, suggestions and recommendations. Some elements of the manuscript's presentation and orientation have been altered in response to the reviewers' recommendations. For ease of reference, manuscript changes have been highlighted in red color. Below is a description of the authors' responses to the editors’ and reviewers' remarks:

| **Required Revision/Editorial Summary** | **Authors’ Response** | **Placement in Revised Manuscript** |
| --- | --- | --- |
| 1. Provide ethics approval documentation for the expert-involvement component or explain why it was exempt under your institutional policy. This is mandatory. | We recognize that providing ethical oversight documentation or justification is a non-negotiable requirement. The study protocol was formally reviewed and deemed **Exempt** from the requirement for full Institutional Review Board (IRB) review by the **Office of the Director (Research & Extension), Khulna University of Engineering & Technology**. The detailed justification for this determination, along with the confirmation of the recruitment period (April 1, 2024, to July 30, 2024) and the collection of **informed written consent** from all participants, is now explicitly included in the **Declaration** section of the manuscript. This confirms adherence to all ethical standards. | **Declaration,** highlighted in red color. |
| 2. Strengthen methodological transparency by fully explaining threshold determination in the Fuzzy Delphi stage and providing clear justification for factor elimination. | We have significantly strengthened the explanation of the Fuzzy Delphi screening process: **Threshold Value (S):** The precise numerical value of the consensus threshold (S), calculated as the average of the defuzzified scores of the initial 26 factors, is now explicitly stated. **Factor Elimination:** We have explicitly listed the four factors that were eliminated in the screening process and confirmed that their CVj score was below the threshold S. | **Section 4.4.4** (Determination of significant factors) and **Section 5** (Case Study), highlighted in red color. |
| 3. Expand or justify the expert sample size and clarify selection criteria. If expansion is not possible, provide a rigorous explanation of sample sufficiency. | Expanding the sample size within the revision window is not feasible due to the study's reliance on highly specialized, judgmental sampling. We have added a more rigorous justification in Section 4.1, emphasizing: **Consensus-Based Approach:** The sample size (n=10) is justified by the Delphi principle of expert consensus, where methodological strength is prioritized over statistical power. **Intentional Diversity:** We re-emphasized that the sample was specifically balanced to cover all four critical functional roles (Clinical, Managerial, Technical, Policy) across different organizational types (Government, Private, Academic) to ensure construct validity and mitigate bias. | **Section 4.1** (Participants & Study Design), highlighted in red color. |
| 4. Reassess the factor set, including Vendor Support, Data Governance, and Digital Literacy, and justify inclusion or exclusion explicitly. | We acknowledge the importance of these factors. We have explicitly addressed their status in the manuscript: **Exclusion Rationale:** We justified their initial exclusion/low ranking by noting that the current market focus in Bangladesh is on foundational issues (management support, readiness) rather than these advanced implementation/ecosystem factors. **Limitation & Future Scope:** We maintained their explicit mention in the Limitations section to be transparent, and strongly advocated for their inclusion in future cross-country research comparing different maturity levels. | **Section 8.1** (Limitations & Future Scopes), highlighted in red color. |
| 5. Revise the manuscript for clarity and readability, correcting language issues throughout and improving flow in the methods and results sections. | We have conducted a final, comprehensive round of language and grammar editing across the entire manuscript. We specifically addressed the instance of poor language quality mentioned in the review (Reviewer 1, Comment 1) and ensured the flow and structure of the Methods and Discussion sections are clearer and more concise, aligning with the standards of PLOS ONE. | Entire Manuscript, highlighted in red color. |

| **Response to Reviewer #1** | | | |
| --- | --- | --- | --- |
| **Reviewer comments** | | **Authors’ response** | **Location in Revised Manuscript** |
| 1. | The manuscript structure and language are affecting the reading flow. The language use at some point is very low (for example, a statement in the methods reads, "In order to fill in the details, we are expected to come up with the first step which is coming up with criteria from a detailed literature review and getting insights from the industry experts."). | Thank you for identifying these issues. We have performed a rigorous language revision on the **Entire Manuscript**, focusing on flow, conciseness, and professionalism. The poor sentence structure cited in the comment has been corrected, and we have ensured clear, academic language is maintained, particularly in the Methods and Discussion sections. | Entire Manuscript, highlighted in red color. |
| 2. | Ethics committee clearance should be obtained for this kind of study to ensure adherence to ethical standards when involving expert opinions. | We recognize that providing ethical oversight documentation or justification is a non-negotiable requirement. The study protocol was formally reviewed and deemed **Exempt** from the requirement for full Institutional Review Board (IRB) review by the **Office of the Director (Research & Extension), Khulna University of Engineering & Technology**. The detailed justification for this determination, along with the confirmation of the recruitment period (April 1, 2024, to July 30, 2024) and the collection of **informed written consent** from all participants, is now explicitly included in the **Declaration** section of the manuscript. This confirms adherence to all ethical standards. | **Declaration,** highlighted in red color. |
| 3. | How are thresholds determined in the Fuzzy Delphi process, and explain properly how the factor elimination is justified to strengthen the credibility of factor selection? | This critical transparency gap has been resolved. We have explicitly stated the **calculated consensus threshold value (S)** and precisely named the **four factors** that were eliminated because their scores were below this threshold. This provides objective justification for the final list of 22 criteria. | **Section 4.4.4** (Determination of significant factors) and **Section 5** (Case Study), highlighted in red color. |

| **Response to Reviewer #2** | | | |
| --- | --- | --- | --- |
| **Reviewer comments** | | **Authors’ response** | **Location in Revised Manuscript** |
| 1. | The study relied on a small sample of only ten experts, which may have limited the generalizability of the results. Authors must expand the expert panel to include a larger and more diverse group of stakeholders to enhance the robustness and generalizability of the factor prioritization. | As detailed in our response to Editorial Revision 3, we have rigorously justified the n=10 sample size based on the **Delphi Consensus Principle** and the **Intentional Diversity** of the functional roles chosen (Table 1), which is methodologically sound for MCDM studies. | **Section 4.1** (Participants & Study Design), highlighted in red color. |
| 2. | Some potentially relevant factors such as Vendor Support and Data Governance were omitted from the analysis due to the initial screening process. Authors must include additional factors such as Vendor Support, Data Governance, and Digital Literacy in subsequent iterations of the model to ensure comprehensiveness. | We justified the exclusion in the current analysis (focus on foundational issues in a developing economy) and explicitly maintained their omission as a **Limitation** that must be addressed by **future cross-contextual research**. | **Section 8.1** (Limitations & Future Scopes), highlighted in red color. |
| 3. | The research was conducted only in the Bangladeshi healthcare sector, which may restrict the transferability of findings to other sectors or countries. Authors should extend the research to other sectors or countries to enable cross-contextual comparisons and validate the model’s applicability. | We agree that context is key. We have added a dedicated discussion on the **Applicable Boundaries** of the framework (e.g., comparison with India) and reinforced the recommendation for **future research to extend the study to other countries and sectors**, using quantifiable metrics (GDP/DAI) to define universal conditions. | **Section 8** (Conclusion), highlighted in red color**.** |
| 4. | The study used a static MCDM approach and did not account for the dynamic interdependencies between factors over time. Authors must adopt dynamic MCDM methods such as Fuzzy DANP or System Dynamics in future work to explore the evolving relationships between factors over time. | We acknowledge the limitation of the cross-sectional, static design. We now explicitly recommend that **future work apply dynamic modeling methods** (e.g., Fuzzy DANP or System Dynamics) to rigorously explore the evolving, time-dependent interdependencies among factors. | **Section 8.1** (Limitations & Future Scopes), highlighted in red color. |
| 5. | Authors must incorporate other MCDM techniques such as AHP, BWM, or TOPSIS in parallel to compare results and enhance the validity of the rankings. | We recognize the value of cross-validation. While performing a parallel MCDM analysis was outside the scope of this initial, foundational work on a novel Dombi LMAW application, we have added a recommendation that **future studies integrate parallel MCDM methods** (AHP, BWM, TOPSIS) to empirically compare rank stability and validate the superiority of the Dombi LMAW weighting in complex fuzzy environments. | **Section 8.1** (Limitations & Future Scopes), highlighted in red color. |
| 6. | The cross-sectional design did not capture longitudinal changes in factor importance or adoption behavior. | This is inherent to the chosen research design. We have confirmed in Section 4.1 that the study used an **observational and cross-sectional analysis**, which determines factor importance at a specific point in time, and this limitation is linked to the need for future dynamic modeling (see response to Comment 4). | **Section 4.1** (Participants & Study Design), **Section 8.1** (Limitations & Future Scopes), highlighted in red color. |

Therefore, the author concluded that, the revised manuscript has addressed all comments of the reviewers and editors. The orientation and presentation of the paper were changed in the revised manuscript to address all reviewers’ and editors’ comments. All changes are highlighted in red text in the revised manuscript. Hence, we look forward to your positive reassessment.
