# Supplementary material for: Strategic prioritization of blockchain diffusion factors by an integrated fuzzy Delphi-Dombi LMAW model: A case study in Bangladesh healthcare sectors
Source: PLoS One. 2026 May 14;21(5):e0348578. doi: 10.1371/journal.pone.0348578 (PMC13175360; doi:10.1371/journal.pone.0348578)
Supplement: Attachment — Submitted filename: Response to Reviewer.docx [file pone.0348578.s004.docx]

**MANUSCRIPT INFORMATION**

**Manuscript Number: PONE-D-25-37578**

**Manuscript Title:** Strategic prioritization of blockchain diffusion factors by an integrated Fuzzy Delphi-Dombi LMAW model: A case study in Bangladesh healthcare sectors.

**Journal:** PLOS ONE

______________________________________________________________________________

We appreciate the constructive feedback provided by the editors and reviewers of PLOS ONE, which helped to make the manuscript better. The authors have prepared a revised manuscript that takes into account all of the reviewers' and editor comments, suggestions and recommendations. Some elements of the manuscript's presentation and orientation have been altered in response to the reviewers' recommendations. For ease of reference, manuscript changes have been highlighted in red text. Below is a description of the authors' responses to the editors’ and reviewers' remarks:

| **Reviewer Comments** | **Authors’ Response** | **Placement in Revised Manuscript** |
| --- | --- | --- |
| 1. Provide ethics approval documentation for the expert-involvement component or explain why it was exempt under your institutional policy. This is mandatory. | We recognize that providing ethical oversight documentation or justification is a non-negotiable requirement. The study protocol was formally reviewed and deemed **Exempt** from the requirement for full Institutional Review Board (IRB) review by the **Office of the Director (Research & Extension), Khulna University of Engineering & Technology**. The detailed justification for this determination, along with the confirmation of the recruitment period (April 1, 2024 to July 30, 2024) and the collection of **informed written consent** from all participants, is now explicitly included in the **Declaration** section of the manuscript. This confirms adherence to all ethical standards. | Participants and Study Design (Line 248-256), highlighted in red text. |
| 2. Strengthen methodological transparency by fully explaining threshold determination in the Fuzzy Delphi stage and providing clear justification for factor elimination. | We have expanded the justification to provide a more rigorous explanation of the threshold determination. We have explicitly defined the **consensus threshold (S = 6.9)** as the arithmetic mean of the defuzzified scores of the initial 26 factors. We have also clarified the selection logic, explaining that the threshold acts as a filter to ensure only factors with high expert consensus are carried forward into the Dombi LMAW prioritization stage, thereby increasing the precision of the final model. **Factor Elimination:** We have strengthened the justification for factor elimination in Case Study Section. Beyond stating the mathematical threshold (S = 6.9), we have added a contextual interpretation for why the four specific factors (OF6, TF7, EF5, and IF4) were eliminated. We clarified that these exclusions reflect the expert panel's focus on foundational organizational and leadership priorities rather than advanced technical or individual behavioral constructs, which is consistent with the current digital maturity level of the Bangladeshi healthcare sector. | **Determination of significant factors** (Line 318-326) and **Case Study** (Line 413-421), highlighted in red text. |
| 3. Expand or justify the expert sample size and clarify selection criteria. If expansion is not possible, provide a rigorous explanation of sample sufficiency. | We have provided a rigorous justification for the sample size (n=10) by grounding it in the **expert-driven consensus principle** of the Delphi method. We have added a citation **(Hsu & Sandford, n.d.)** to support the methodological standard that a panel of 10-15 experts is sufficient for stable consensus in this framework. Furthermore, we have clarified that our purposive sampling ensured complete coverage of all four critical functional roles (Clinical, Managerial, Technical, Policy) to maintain construct validity. | **Participants & Study Design** (Line 229-245), highlighted in red text. |
| 4. Reassess the factor set, including Vendor Support, Data Governance, and Digital Literacy, and justify inclusion or exclusion explicitly. | We acknowledge the importance of these factors. We have explicitly addressed their status in the manuscript: **Exclusion Rationale:** We justified their initial exclusion/low ranking by noting that the current market focus in Bangladesh is on foundational issues (management support, organizational readiness) rather than these advanced implementation/ecosystem factors. **Limitation & Future Scope:** We maintained their explicit mention in the Limitations section to be transparent, and strongly advocated for their inclusion in future cross-country research comparing different maturity levels. | **Limitations & Future Scopes** (Line 678-685), highlighted in red text. |
| 5. Revise the manuscript for clarity and readability, correcting language issues throughout and improving flow in the methods and results sections. | We have conducted a final, comprehensive round of language and grammar editing across the entire manuscript. We specifically addressed the instance of poor language quality mentioned in the review and ensured the flow and structure of the Methods and Discussion sections are clearer and more concise, aligning with the standards of PLOS ONE. | Entire Manuscript |

Therefore, the author concluded that, the revised manuscript has addressed all comments of the reviewers and editors. The orientation and presentation of the paper were changed in the revised manuscript to address all reviewers’ and editors’ comments. All changes are highlighted in red text in the revised manuscript. Hence, we look forward to your positive reassessment.
